# The Association Between Body Mass Index (BMI) and Sleep Duration: Where Are We after nearly Two Decades of Epidemiological Research?

**DOI:** 10.3390/ijerph16224327

**Published:** 2019-11-06

**Authors:** Victoria Garfield

**Affiliations:** MRC Unit of Lifelong Health and Ageing at UCL, Department of Population Science and Experimental Medicine, Institute of Cardiovascular Science, University College London, 1-19 Torrington Place, WC1E 7HB London, UK; v.garfield@ucl.ac.uk; Tel.: +44-20-3549-5589

**Keywords:** sleep duration, body mass index, obesity, epidemiology, causality

## Abstract

Over the past twenty years we have seen a vast number of epidemiological studies emerge on the topic of obesity and sleep duration, with a focus on body mass index, as it is easy and cheap to measure and analyse. Such studies largely observe that cross-sectionally a higher BMI is associated with shorter sleep and that in longitudinal studies shorter sleep duration is associated with increases in BMI over time, but some research has found no relationship between the two. This narrative review is not exhaustive, but appraises the literature on sleep duration and BMI from perspectives that have previously been unexplored in a single paper. As such, I discuss research in these important areas: bidirectionality, objective vs. subjective sleep duration, how meaningful the effect sizes are and how we have begun to address causality in this area. From the evidence appraised in this review, it is clear that: (i) there is some modest evidence of a bidirectional relationship between BMI and sleep duration in both children and adults; (ii) objective measurements of sleep should be used where possible; (iii) it remains difficult to confirm whether the effect sizes are conclusively meaningful in a clinical setting, but at least in adults this so far seems unlikely; (iv) to date, there is no solid evidence that this relationship (in either direction) is in fact causal. In the near future, I would like to see triangulation of these findings and perhaps a move towards focusing on distinct aspects of the relationship between obesity and sleep that have not previously been addressed in detail, for various reasons.

## 1. Introduction

The first large-scale epidemiological study to explore obesity/body mass index (BMI) and sleep duration was published in 2000 and suggested that being obese was associated with less sleep [1]. Since then hundreds of studies investigating the association between BMI and sleep duration have been published in both paediatric and adult samples. The majority of studies use BMI, as it is an adiposity measure that is commonly and easily collected in a wide range of research settings, and it is simple to analyse and interpret. Meta-analyses and systematic reviews over the years have largely observed that, both cross-sectionally [2,3,4,5] and longitudinally [3,4,6,7,8,9,10,11], shorter sleep is associated with higher BMI in children and adults. However, some studies have observed no cross-sectional [12,13,14,15,16,17,18,19,20] or prospective relationship [21,22,23,24] between BMI and sleep duration. The aim of this review is not to provide an exhaustive appraisal of the epidemiological literature to date. However, this review will try to provide readers with a different perspective of the evidence in this area. Thus, I will review studies that have not traditionally been mentioned in most systematic reviews and meta-analyses of BMI and sleep duration. The topics that I will give careful consideration are: bidirectionality in the association between BMI and sleep duration; objective vs. subjective sleep duration; reliability and size of effects; and causality.

## 2. Bidirectionality

### 2.1. Children

**Figure 1:**
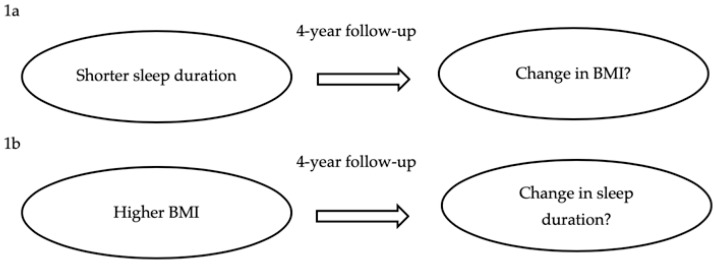
Illustration of the potential bidirectional relationship between body mass index (BMI) and sleep duration, using a 4-year follow-up as an example. This figure exemplifies the potential bidirectional association between BMI and sleep duration; a 4-year follow-up was used, as this was what we used in a recent bidirectional study of older adults [27]. The main hypotheses were around shorter sleep duration and how this may be associated with change in BMI over time (**1a**), and the reverse association focused on higher BMI at baseline and its association with changes in sleep duration over time (**1b**).

In 2010, Vgontzas and colleagues published an important commentary [25], in which they noted that the majority of observational longitudinal studies had explored only the association from sleep duration to BMI. However, it seemed very plausible that changes in BMI could be associated with changes in duration of sleep over time (Figure 1). Until 2016, there had been only two published studies that explored the potential bidirectional association between BMI and sleep duration, both of which used paediatric data [23,26].

The study by Hiscock et al. [23] found no prospective association between BMI and sleep duration, in either direction, in a national sample of ~4000 Dutch children aged between zero and seven years. In cross-sectional analyses, however, they observed that at ages six/seven years children who were obese slept for 30 min less than those in the underweight, normal weight and overweight groups. These findings contrasted with numerous epidemiological studies, and this could be due to at least two reasons. Firstly, the authors used 24-h time diaries to collect data on sleep duration whilst the majority of previous research at the time had used parent-reported sleep duration, and it is this measure—rather than time diary reports—that predicts obesity. Therefore, this could mean that their results were in fact more accurate than studies that use parent-reported sleep duration. However, the study used a bespoke measure to ascertain sleep, which has not been used in other studies and for which no validity or reliability measures were provided, which may mean that the findings are uncertain. Secondly, it is possible that the relationship between short sleep and paediatric obesity develops slightly later, which is in line with their finding from the six-to-seven-year-old children. One hypothesis could be that the relationship between shorter sleep and obesity is via eating behaviour, and as such, a child would need to consume excessive amounts of food over a certain number of years (as a consequence of short sleep), which would then lead to increased weight/risk of obesity later. It is also possible that children who eat more when they are tired are likely to have more autonomy over what, when and how they consume food, which is a privilege that usually comes as children become older. This is supported by epidemiological research in which shorter sleep in early life (16 months) is associated with greater energy intake, but this effect emerges prior to the association with weight [28]. More specifically, this study by Fisher and colleagues showed that there was no relationship between sleep duration and weight in a sample of 1303 British children, but that the association between energy intake and sleep duration was strong [28].

Collings et al. [26] also investigated the bidirectional relationship between BMI and sleep duration. They analysed data from 776 South Asian and 562 White children from the Born in Bradford-1000 (BiB-1000) cohort at ages 12, 18, 24 and 36 months of age. Their results showed that the association between BMI and parent-reported sleep duration was significant in both directions in South Asian children, but the findings were null in White children. In the South Asian children BMI had a two-to-threefold larger effect on sleep duration, rather than the other way around (i.e., sleep duration as the exposure). Finding that BMI and sleep duration were associated in both directions in South Asian children requires replication in a larger and independent sample. The findings in White children are comparable with those of Hiscock’s study, even though Collings used parent-reported sleep duration as opposed to sleep diaries.

A much more recent study also explored the bidirectional association between BMI and subjective sleep duration, using data from a Dutch population-based-cluster randomised control trial of 2308 children aged between 0 and 3 years [29]. Their findings showed that sleep duration at 6 or 14 months of age was not associated with BMI z-score at 14 or 36 months, respectively. However, higher BMI z-scores at 6 months were related to approximately 8 min less sleep, but no such effect was observed between BMI z-scores at 14 months in relation to sleep duration at 36 months. This result is in contrast with the two bidirectional studies by Klingenberg et al. [17] and Collings et al. [26], as Wang and colleagues did find an association between BMI and changes in sleep duration in young children of White ethnicity. Whilst Collings [26] observed a relationship between BMI and change in sleep duration in South Asian children only, the authors of the Dutch study suggested that one plausible reason for a lack of an effect in the non-White Dutch participants could be that they are less homogenous and are more likely to be of mixed ethnicity.

Two of these three studies used parent-reported sleep duration [26,28], whilst the other study used 24-h sleep diaries [17]. There have been no paediatric bidirectional studies of BMI and sleep duration that have used objective sleep duration, and therefore, these findings have yet to be replicated or refuted using these sorts of measurements. There have been no studies that have examined the bidirectionality of this relationship in adolescents, and as such, this is not covered here.

Importantly, when it comes to the relationship between BMI and sleep duration, one possibility is that there may be confounding by specific body composition measures, but not due to fatness. The fact that BMI cannot distinguish muscle mass from fat mass is also important to consider, and as such, future research should consider body fat percentage, particularly in children. For example, having greater muscle mass means individuals have a higher BMI, but this is not due to fat. Longitudinal research in 244 children from New Zealand showed that objectively measured sleep duration at ages 3 to 5 years (an average of the two was taken) was negatively associated with fat mass index at age 7 years in multiply adjusted models (sex, maternal education, maternal BMI, income, ethnicity, birth weight, smoking during pregnancy, physical activity, TV viewing, fruit/vegetable intake and non-core foods intake) [30]. The authors also found a negative prospective association between sleep duration and BMI, but their findings suggested that these differences in body weight (between children with varying hours of sleep) were accounted for by increased fat mass deposition.

### 2.2. Middle-Aged and Older Adults

Along with colleagues, in 2017, we published the first study that investigated the bidirectional association between BMI and sleep duration in adults, which focused on adults over the age of 50 years [27]. We used data from the English Longitudinal Study of Ageing (ELSA)—described in detail elsewhere [31]—to investigate whether BMI might predict changes in sleep duration, and/or vice versa, over a four-year follow-up. Our findings showed that over the follow-up period and after adjustment for multiple confounders (demographics, health behaviours, health problems), BMI was associated with very small decreases in sleep duration, with an effect size of (unstandardized coefficient from linear regression) −0.42 min (this will be revisited later in Section 4). We observed no association in the opposite direction, and thus sleep duration at baseline was not related to changes in BMI over four years. However, like any other study, ours had limitations, of which the main ones were: the use of self-reported sleep duration, there was no measure of napping (important for older adults) and we had no data on whether any of our participants had sleep apnoea. Much more recently, one other bidirectional analysis of this association in middle-aged and older adults was published, which was the first to use objective sleep data in this context [32]. The authors found that prospectively, longer sleep duration was associated with a lower BMI and additionally, that a one-unit increase in BMI (kg/m^2^) was related to 1.2 min shorter sleep. Although this effect size is three times greater than the 0.42 min that we observed in our study, it is difficult to say that this is meaningful in terms of clinical translation. There have been no studies that have examined the bidirectional relationship between BMI and sleep duration in young adults, and therefore it is not covered here.

## 3. Objective vs. Subjective Sleep Duration

### 3.1. Brief Overview

Sleep duration can be measured in different ways, which can be broadly classified as subjective and objective methods. Subjective sleep duration usually involves asking how many hours people sleep per night, whilst objective sleep research either monitors participants in a laboratory setting or asks them to wear a device that tracks their sleep duration. The most common objective sleep duration methods are polysomnography (PSG) and actigraphy. PSG is considered the gold standard for diagnosing certain sleep disorders [33]. On the other hand, an actigraph is a device that is usually worn on the wrist or waist, and a questionnaire is administered along with it. The questionnaire aids interpretation of the data collected by the actigraph, as it asks questions about whether people are equally as active/inactive and whether they were ill during the time that they wore the actigraph [34].

### 3.2. Subjective Sleep Duration

The main advantages of self-reported sleep duration measures are that they are inexpensive and easy to administer, as often one to two questions can be asked as part of a larger battery of tests; they are simple to code and to analyse, as a typical question asks about the number of hours that people spend asleep. Importantly, most studies use self-reported measures of sleep duration because of these advantages, and therefore making comparisons across studies is straightforward. These measures also have criterion validity, as research suggests that both subjective short and long sleep duration are associated with coronary heart disease (CHD) [35], stroke [35], type-2 diabetes [36] and mortality [37]. However, subjective sleep duration has its limitations. There is potential for measurement error, as some people misreport the number of hours they sleep. For example, they may not know and might guess their sleep duration, they may have irregular sleep patterns making it difficult to estimate, or they might not be keen to provide a true report if they have atypical sleep durations. Some people may report the number of hours they sleep from the time they actually go to bed, rather than the time they fall asleep. This time between full wakefulness and sleep onset is known as sleep latency, and is different from sleep duration. Importantly, though, most normal sleepers who do not suffer from a sleep disorder (particularly insomnia) tend to report similar sleep duration estimates to those observed in the laboratory under PSG [38]. Finally, the correlation between objective and subjective sleep duration has been reported to be around 0.45 [39], whilst another study found that 34% of participants had a discrepancy of one hour or more between sleep diaries and actigraphy [40], and a more recent report found that the correlation between subjective and actigraphic sleep duration was only 0.3 [41].

### 3.3. Objective Sleep Duration

Actigraphy has been used in research for more than twenty-five years to assess sleep and wakefulness states [42]. An actigraph is usually worn on the wrist of the non-dominant arm and continuously detects and records body movement (largely acceleration), and stores the information for extended periods of time [43]. However, waist-worn actigraphs can also be used for measuring sleep parameters, alongside the detection of movement and activity. It is now also possible to collect data using specific applications on smartphones and smart watches. Actigraphy is popular for determining patterns of sleep and circadian rhythms and, unlike PSG, does not demand that people spend time in a laboratory. Actigraphs can be worn for several weeks at a time, are more cost effective for data collection in large-scale studies and are less invasive than PSG, particularly when collecting sleep data from infants and elderly adults [44]. Although PSG is considered the gold standard for the measurement of sleep/wake behaviours, actigraphy can provide more reliable data, as they are collected over several days rather than over one or two nights in a laboratory [44]. Actigraphy may also be more ecologically valid, as it measures habitual sleep in people’s usual environment, rather than in a lab, which is an unnatural setting to sleep in and thus could affect sleep. Whilst data are being recorded via actigraphs individuals can continue with daily activities and remain in their natural sleep environments. Actigraphs are now used to diagnose and evaluate insomnia, extreme sleepiness, restless legs syndrome and circadian rhythm disorders [44]. When using actigraphy, algorithms are used to calculate sleep and wake periods, based on people’s activity patterns [45]. However, a sleep diary is usually administered alongside it, as an actigraphs are not able to differentiate between sitting, lying motionless and sleep [46].

However, as with any other measurement, actigraphy has its disadvantages. Actigraphs are unable to reliably differentiate sleep stages, are more expensive than subjective sleep duration measures, have at times shown poor specificity when measuring sleep duration and may sometimes over- or underestimate sleep duration. For example, a study by Paquet and colleagues [46] showed that actigraphy had overall less than 50% specificity in determining sleep and also overestimated sleep duration when compared with PSG. In another study of sixty-eight women, actigraphy underestimated sleep durations by an average of 68 min in participants who slept for less than five hours [44]. This could be due to sleep disturbances leading to increases in nocturnal movement and therefore causing the actigraph to underestimate sleep duration.

### 3.4. Verdict on Subjective vs. Objective Methods

Objective methods for measuring sleep duration are rapidly becoming less expensive, particularly with the advent of (as mentioned above) collection via smartphones and smartwatches. However, the majority of longitudinal epidemiological cohorts have understandably yet to implement this sort of data collection for sleep duration (as well as other sleep parameters) across all participants, due to costs and funding priorities. The UK Biobank, which is a large epidemiological study of 500,000 UK adults aged between 40 and 70 years [47], has collected actigraphy in approximately 100,000 participants. To date, this is the largest sample of individuals with actigraphic sleep measurements, and due to the breadth of phenotypic data measured in UKB participants we can expect there will be a wide range of published studies using these data in relation to measures such as BMI and many, many other important traits.

Although it is fundamental to acknowledge that objective measurements are usually superior and ought to be used where possible, as mentioned earlier, subjective sleep duration has its advantages. It is also important to note that in the analysis of within-person observational data of sleep duration, poor agreement between subjective and objective sleep measures is likely to be less of an issue. This is because the error terms associated with the measurement remain consistent and are thus unlikely to invalidate the findings.

## 4. Size, Consistency and Clinical Significance of Effects

The focus in this section is on effect sizes that have emerged from meta-analyses of prospective studies of sleep duration and obesity (usually derived from BMI cut-off points), rather than cross-sectional studies. This is because longitudinal data can at least begin to provide us with some evidence for a particular direction of effect and is thus more meaningful than cross-sectional data—particularly in the context of a topic that has been researched as much as BMI and sleep duration.

### 4.1. Size and Consistency of Effects

#### 4.1.1. Children

Five meta-analyses have focused on the prospective relationship between sleep duration and BMI (in that direction specifically) in children over the past decade [7,8,9,10,11]. The key effect sizes reported by less-recent meta-analyses were odds ratio (OR) = 2.15 (95% confidence interval (CI) = 1.64–2.81) [7], OR = 1.76 (95% CI = 1.39–2.23) [8], relative risk (RR) = 1.30 (95% CI = 1.20–1.42) [9], OR = 1.71 (95% CI = 1.36–2.14) [10]. However, Miller et al. published the most recent meta-analysis of sleep duration and obesity [11]. Due to heterogeneity, the authors reported separate effect sizes for different ages: in infancy, RR = 1.40 (95% CI = 1.19–1.65); in early childhood, RR = 1.57 (95% CI = 1.40–1.76); and in middle childhood, RR = 2.23 (95% CI = 2.18–2.27).

Whilst the above-described inconsistencies in effect sizes across meta-analyses are noteworthy, it is also important to remember that there are now dozens of studies that have explored the relationship between sleep duration and BMI/obesity, and they tend to differ in terms of population under study, sample size and inclusion of confounders. Amongst the dozens of paediatric studies there has been no attempt to try to understand whether the association between sleep duration and BMI/obesity is causal, or whether the opposite relationship might be causal. Moreover, the majority of meta-analyses and reviews have focused on only one direction of association, that is, from sleep duration to BMI, rather than the other way around.

#### 4.1.2. Adults and Older Adults

There has been only one meta-analysis of the prospective association between sleep duration and obesity (ascertained using BMI cut-off points) in adults [6]. This meta-analysis published in 2014 yielded a pooled OR of 1.25 across eight studies with a total of over 100,000 participants. This effect is smaller than any of those reported in the paediatric meta-analyses and is thus supportive of evidence which has suggested that the relationship between sleep duration and BMI weakens with age [48,49]. Related to and in support of this point about effects that diminish as a function of age is the finding reported earlier on the diminutive effect that we observed in adults over the age of 50 (i.e., for every unit increase in BMI, sleep duration changed by 0.42 min, and no longitudinal association was found in the opposite direction) [27].

### 4.2. Clinical Significance of Effects

It is difficult to reach a consensus about how clinically significant the effects reported above are and as such, how health professionals ought to interpret them and advise patients accordingly. There are clearly differences by age, and the largest effect to date is that of RR = 2.23 observed in middle childhood in the latest meta-analysis by Miller and colleagues [11]. It is important to note that this estimate was across three studies [50,51,52] with a total sample size of 3005, all of which were North American (two from the USA and one from Canada). Thus, perhaps before we recommend that in this age group (9–12 years) medical professionals pay special attention to the weight/BMI of those who sleep for less than the recommended number of hours (9–11 h) this particularly large effect requires robust replication, and potentially some attempt to determine whether these effects are in fact causal in nature. Additionally, these three studies differed substantially in the confounder adjustments made in their analyses, which is likely to have had an effect on the individual study results.

Whilst we have a rapidly increasing ageing population, it is important that we remain focused on the main health problems and comorbidities that this age group faces, so that we can design appropriate interventions and ensure that we provide older adults with the best possible quality of life. If sleep duration in relation to weight/BMI is not a prominent target for intervention in this age group, then it is perhaps best that we focus our efforts elsewhere when it comes to older adults. Importantly, though, the very small effect observed in the only bidirectional study of BMI and sleep duration in this age group [27] deserves replication across different ageing cohorts, both within and without similar cultural settings (e.g., cross-cultural comparisons).

## 5. Causality

### 5.1. Findings from Mendelian Randomisation (MR) Studies

**Figure 2:**
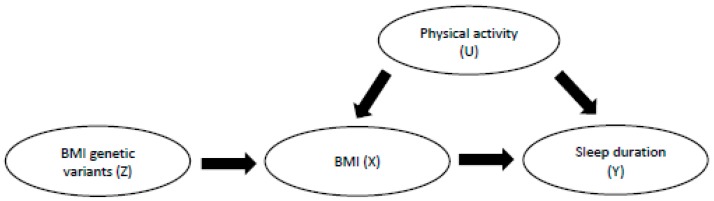
Directed acyclic graph (DAG) to illustrate the Mendelian randomisation paradigm. Z denotes the genetic variants (or single nucleotide polymorphisms, SNPs) that have been associated with BMI in genome-wide association studies (GWAS) and that are used in this framework as instrumental variables or proxies of our exposure under study [53], BMI (X). Physical activity (U) represents an example of a common unobserved confounder of the relationship between BMI (X) and our outcome of interest, sleep duration (Y).

The ascertainment of whether a relationship is causal is not trivial, and different methods exist to aid in our understanding of whether an association between two traits might be causal or not. Although the widely accepted gold standard for determining causality is the randomised control trial (RCT), this kind of study is often expensive and time-consuming in its design and implementation. Thus, in 2003, Davey-Smith and Ebrahim [53] wrote a seminal paper in which they proposed that Mendelian randomisation (the random assortment of alleles—different forms of a gene—from parents to offspring) could be used in aetiological epidemiology to investigate causality. It is an approach that can be employed to try and determine whether an association that has been relatively well established in the epidemiological literature may be causal [53]. Figure 2 illustrates the MR paradigm, in which common genetic variants (single nucleotide polymorphisms—SNPs) are used to instrument the association between a given modifiable exposure and an outcome of interest. These common genetic variants are used in MR, as they have certain unique properties: an individual’s genome is determined at conception and remains stable throughout the life course (no problems with reverse causality) and are unlikely to be associated with common confounders of the association under study (helps deal with issues of unobserved confounding that are usually present in observational studies) [53]. The core assumptions of MR are threefold: (i) there must be a robust association between the exposure of interest and its relevant genetic variants (usually from published and replicated genome-wide association studies (GWAS)); (ii) there should be no direct association between the genetic variants for the exposure and the outcome of interest; (iii) in relation to the above point about confounding, there should be no association between the genetic variants for the exposure and common confounders of the exposure–outcome relationship under study.

Although MR has now been applied to countless well-known observational associations, until 2016, there had been no attempt to use it to investigate whether the relationship between BMI and sleep duration, or sleep duration and BMI, might in fact be causal in nature. Jones and colleagues [54] were the first to publish an MR study of BMI and subjective sleep duration in ~127,000 adults and found no evidence of a causal relationship between the two. The authors implemented what are known as “conventional MR” analyses as well as additional analyses to test the assumptions of MR, and all of their findings pointed towards no causal association in what is considered a very large sample. It seems plausible that the authors would have explored causality in the opposite direction (sleep duration → BMI), but they did not. The likely reason for this is that they only found three common genetic variants associated with subjective sleep duration in their study, which would violate the first core MR assumption, mentioned above. A more recent study that did in fact perform the first MR of sleep duration (using an improved 78-SNP instrument from newly-discovered loci) on BMI observed no causal relationship between the two in over 400,000 adults [55].

Based on the use of MR, it would appear that there is no causal association between BMI and subjective sleep duration, or between sleep duration and BMI in adults. Thus, whilst we could jump to conclude that this clearly means that what we have observed for almost two decades are mere associations and there are no causal effects, it is important to remember that MR is not a panacea for every exposure–outcome relationship and is just one of many tools to help us ascertain causality.

### 5.2. Findings from Experimental Studies

Until recently, the most widely accepted causal explanation for the relationship between sleep duration and BMI (as well as increased risk of obesity) was via energy intake. However, in recent years the possible effects of sleep deprivation on energy metabolism have been investigated in experimental studies. I do not discuss these at length here, as this has already been done in high-quality reviews [56,57] and the focus of the present paper is not energy expenditure, but sleep duration and BMI as exposures/outcomes. However, it is important to note that total energy expenditure (TEE) is increased in adolescents during restricted sleep [57], which we might expect to decrease weight if no other compensatory factors (e.g., via sleep homeostasis, eating behaviour or physical activity—all of which could counter the effects of sleep restriction) are implicated.

Randomised control trials (RCTs) of body weight outcomes in humans have also been thoroughly systematically reviewed and meta-analysed and there appears to be no solid evidence to suggest a causal effect of sleep duration on obesity-related outcomes such as BMI [57]. However, only eight studies were included in this review/meta-analysis, and they differed hugely in terms of sample size, intervention duration and type, study design (crossover RCT vs. simple RCT vs. group RCT), age range of participants and the primary outcome measured [57]. 

### 5.3. Consensus on Causality

Before we reach a consensus about whether BMI causes changes in sleep duration, we must triangulate these observations using other well-established causal inference methods [58]. These approaches are detailed in a paper by Lawlor and colleagues [58], but briefly, some examples are: negative control studies, sibling or twin comparisons, and cross-cultural comparisons, amongst others. Crucially, the MR studies published to date on BMI and sleep duration and vice versa have all been carried out in adults, and it is fundamental that in the near future we exploit large-scale consortia data to determine whether these effects might be causal in children and/or adolescents. One important rationale for this is that the effects of the BMI SNPs on BMI tend to be stronger in younger age groups [59,60]. In addition, although individuals’ DNA remains unchanged throughout the life course, differential levels of gene expression have been linked to disease states and cellular responses [61].

To date, experimental studies largely suggest that sleep restriction leads to increased food (energy) intake, which over time may result in weight gain if there is no net compensation. Changes in appetite hormones such as increased ghrelin (that stimulates appetite) and decreased leptin (that suppresses appetite) are also well-known consequences of short sleep [62]. However, it is unclear to what extent the degree of net effect is observed over longer periods of time, as none of these studies have had durations longer than three weeks [57]. Furthermore, these studies have limited generalizability for several reasons: they have mostly been conducted in small samples, with the largest sample size in the region of 15 participants; these studies have not analysed uniform outcomes, making it difficult to draw direct comparisons between them; and intervention periods have typically been too short to assess any real long-term impact of sleep restriction on TEE and on any subsequent weight change. Additionally, there have been approximately fifteen studies of this kind, which precludes global conclusions; and crucially, these types of studies are carried out in laboratory settings, which do not resemble real-life, free-living conditions.

## 6. Conclusions 

The last two decades have yielded a very large number of epidemiological studies on the relationship between sleep duration and BMI/obesity, both in children and adults. While it is widely accepted that this association is an important one (and as such, studies in this area continue to receive attention), we must consider certain aspects of these studies that have so far not received much mention. These include the bidirectionality of the association between BMI and sleep duration, objective vs. subjective duration of sleep, the size and clinical meaning of effects and whether these effects are causal or not in nature. Related to this point, another very informative type of study would be one that has data on objective sleep duration, body fat and composition, energy consumption and expenditure, and that tracks these participants over as many years as possible.

Whilst the majority of research has used BMI, this is often not the best measure of adiposity and thus we should perform more research that aims to understand both bidirectionality and causality between other measures of adiposity (e.g., waist–hip ratio, waist circumference, etc.), body composition and sleep duration.

Overall, the epidemiological evidence suggests that the relationship between BMI and sleep duration in children is stronger than in adults, as we observe a decrease in the effect size as a function of age, such that these effects in older adults appear to be very small. In addition, the effects found in observational meta-analyses have not yet been replicated using causal methods, and further studies that employ a wider range of causal methods are required. We should also shift our focus away from cross-sectional studies in this area, of which there are now hundreds, and conduct more research that is longitudinal and incorporates causal inference methods other than Mendelian randomisation to further understand the nature of the relationship between BMI and sleep duration. Of course, the incorporation of other measurements, such as blood biomarkers and multi-omics measures (metabolomics, proteomics, epigenomics, etc) would also be immensely valuable here, but these studies are still lacking in the realm of BMI and sleep duration.

This review has provided insight (albeit not extensive) into the above-mentioned aspects of the association between sleep duration and BMI. In doing so, it has given a few recommendations on where we might direct research in this area to help establish in more depth what might underlie this association that is often taken for granted.
